# Improvement of China’s Shan-Shui Initiative: strategic pathways for its sustainable development

**DOI:** 10.1093/nsr/nwag215

**Published:** 2026-04-08

**Authors:** Yanjun Shen, Shengwei Zhang, Tieming Liu, Yajun Li, Jianbing Peng

**Affiliations:** Department of Geological Engineering and Geomatics, Chang’an University, China; Key Laboratory of Ecological Geology and Disaster Prevention, Ministry of Natural Resources, China; Shaanxi Provincial Department of Natural Resources, Headquarters of Qinling North Foot Shan-Shui Ecological Protection and Restoration Project, China; Department of Geological Engineering and Geomatics, Chang’an University, China; Key Laboratory of Ecological Geology and Disaster Prevention, Ministry of Natural Resources, China; Shaanxi Provincial Department of Natural Resources, Headquarters of Qinling North Foot Shan-Shui Ecological Protection and Restoration Project, China; Beijing Municipal Commission of Urban Planning and Natural Resources, Headquarters of Shan-Shui Ecological Protection and Restoration Project in Western Ecological Barrier Area of the Capital, China; School of Engineering and Technology, China University of Geosciences, China; Department of Geological Engineering and Geomatics, Chang’an University, China; Key Laboratory of Ecological Geology and Disaster Prevention, Ministry of Natural Resources, China; Shaanxi Provincial Department of Natural Resources, Headquarters of Qinling North Foot Shan-Shui Ecological Protection and Restoration Project, China; School of Engineering and Technology, China University of Geosciences, China

## Abstract

This study summarizes decade-long achievements of China's Shan-Shui Initiative (SSI) and proposes future NbS-oriented pathways by dissecting key challenges.

In a literal sense, *Shan-Shui* refers to mountains and rivers. However, its connotation extends far beyond topography, representing an ancient Eastern ecological philosophy that advocates for the symbiotic harmony between humanity and nature. Rooted in this time-honored philosophy, China’s *Shan-Shui* Initiative (SSI) was launched in 2016, which institutionalized an integrated restoration model for diverse ecosystems—mountains, rivers, forests, farmlands, lakes, grasslands, and deserts—aligning with the UN 2030 Agenda and the Kunming–Montreal Global Biodiversity Framework. Departing from traditional single-goal paradigms, the SSI embodies a systematic ‘community of life’ governance concept. By late 2024, 52 projects across 29 provinces have restored ∼8 million ha, strategically positioned at national ecological security nodes within the *Three Zones and Four Belts* (TZFB) framework that is China’s core national ecological security blueprint, comprising three key eco-zones and four major shelterbelts to guide nationwide ecosystem protection and restoration [1,2].

The SSI strives to address legacy issues from 50 years of rapid economic growth, including soil erosion, forest-grass degradation, water pollution, and biological habitats fragmentation. The implementation of SSI has transformed regional land-use patterns and driven positive shifts in ecosystem services. Assessments of early projects (2016–2018) demonstrate significant gains in ecosystem services: soil conservation increased by 6.5%, water conservation by 14.3%, carbon storage by 13.7%, habitat quality by 0.7%, and sand-fixation capacity by 5.07% (Fig. 1) [3].

**Figure 1.**
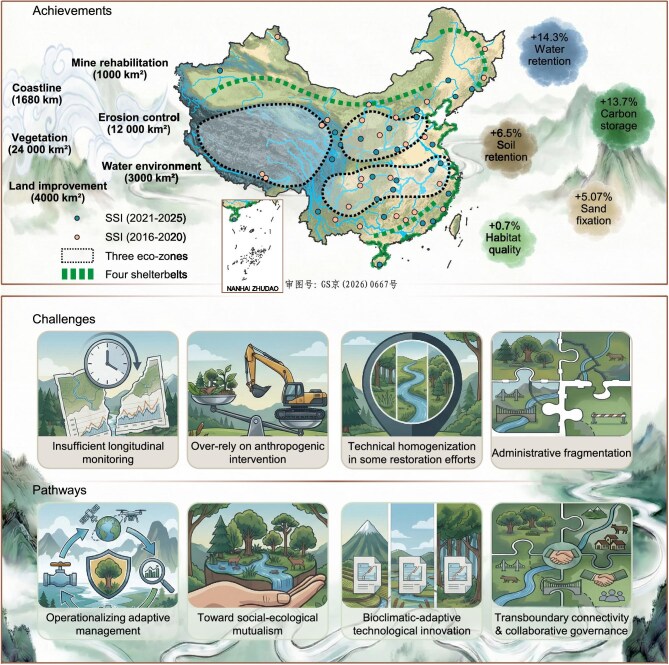
Achievements, challenges, and strategic pathways of China’s SSI. Data source: Ministry of Natural Resources, China [1].

Guided by the ‘lucid waters and lush mountains are invaluable assets’ philosophy, the SSI synchronizes restoration with regional economic strategies, deploying 22 projects in Western China. The promotion of ‘eco-plus’ industries has operationalized the conversion of ecological assets into tangible socioeconomic dividends. By aligning industrial growth with ecosystem service valuation, the SSI has catalyzed regional revitalization across Northeast and Western China, effectively reconciling environmental stewardship with economic prosperity. The 25 SSI projects implemented from 2016 to 2020 generated employment opportunities for more than 400 000 people living in poverty, with an average annual per capita income increase of nearly 6000 yuan (From the Ministry of Finance of PRC, https://bj.mof.gov.cn/ztdd/czysjg/jyjl/202303/t20230328_3875191.htm). Till to the end of 2024, the SSI had created over 1.2 million employment and helped 20 poverty-stricken counties achieve ecological poverty alleviation (https://cloud.kepuchina.cn/h5/detail?id=7389787439753531392). Consequently, the SSI was recognized as a UN ‘World Restoration Flagship’ in 2022, providing a scalable model for balancing ecological protection with economic development [4].

While the SSI has achieved remarkable accomplishments over the past decade, it has also presented some challenges that need improvement in certain projects or stages. Based on the new starting point of its second decade, we summarize the common challenges in SSI implementation, in collaboration with colleagues from the Ministry of Natural Resources (MNR) and the Ministry of Ecology and Environment (MEE), hoping to provide references for the future advancement of the SSI:

1. *Evaluation dimensions are limited to completion milestones, with insufficient longitudinal monitoring.* The prevailing framework in these early projects tends to focus on short-term metrics, such as vegetation growth and land consolidation area, rather than emphasizing the longitudinal monitoring—a dynamic monitoring model based on long-term time-series observations that tracks the continuous changes of ecosystem structure and function over decades—of the long-term successional dynamics of ecosystem functions [5]. This may occasionally result in ‘surface-greening bias’ at the acceptance stage, while substantive gains in biodiversity and carbon sequestration remain scientifically unverified. Some early SSI projects (2016–2020) lacked unified national standards, leading to fragmented protocols that prioritized surface revegetation over longitudinal metrics like soil microbial restructuring or heavy metal migration. For instance, one SSI project located in the Loess Plateau primarily relied on verifying new farmland area and slope compliance; however, while the conversion of sloped land into terraces successfully mitigated soil erosion, it simultaneously triggered a decline in agro-ecosystem biodiversity. Furthermore, in the absence of sustained monitoring in certain projects, the essential transition from anthropogenic intervention to autonomous natural succession lacks sufficient empirical verification.
2. *Engineering design in some projects tends to over-rely on anthropogenic intervention rather than nature-restoration-led orientation.* Certain projects exhibited a pronounced over-engineering tendency, such as high-intensity artificial afforestation even in areas with high ecological resilience. A typical case is the land consolidation project in the karst rocky desertification area of southwest China, where large-scale artificial terracing was carried out on steep slopes with thin soil layers, which did not only to control soil erosion but also destroyed the original micro-topography suitable for the growth of native herbaceous plants, and aggravated vegetation degradation. This ‘hard’ measures escalate costs and risks ecological simplification [6]. Excessive intervention, without understanding natural succession laws, may breach ecosystem self-recovery thresholds. A shift from landscape to functional restoration, prioritizing natural forces with targeted human assistance, is urgently needed.
3. *Addressing technological homogenization in some regional restoration efforts.* A trend of technological homogenization in restoration has been observed in some regions, characterized by a lack of differentiation across key ecological regions. Despite China’s immense geomorphological complexity and the distinct restoration goals of specific ecosystems, existing technical approaches remain homogenized, failing to fully encapsulate the functional nuances of the TZFB framework [7]. The integration of localized technologies and engineering paradigms targeting extreme habitats—such as alpine, arid, and humid-heat environments—has yet to be fully realized. For instance, some early SSI projects in northern China introduced southern evergreen broad-leaved species to enhance ‘greenness’, disregarding the bio-climatic laws of local temperate deciduous forest. Similarly, one coastal SSI project relied on conventional models of hard revetment and artificial beach in specific segments, ignoring the unique requirements of silty or coral reef coastlines.
4. *Administrative fragmentation may hinder robust cross-regional governance in some trans-boundary contexts.* According to the managements implemented in the past, most of these SSI projects would clearly designate isolated county- or city-level administrative units as the main responsible entities [1]. However, with the increasing advancement of SSI projects towards integrated protection and restoration of river basins and mountains in recent years, the layout of projects across administrative divisions has become an inevitable trend. Due to the public-good nature of ecological products, market-based mechanisms often fail to achieve equitable reciprocity in cross-jurisdictional ecological compensation, and this problem is particularly prominent in SSI projects involving multiple cities. For instance, in a currently implemented SSI project in the key ecological zone of the Yellow River, upstream cities tend to control soil erosion through high-intensity engineering measures, while downstream cities focus more on prioritizing the improvement of the water environment. This discrepancy directly leads to inconsistencies in restoration goals among different jurisdictions. In addition, due to the lack of a unified ecological compensation mechanism, upstream regions bear the main costs of ecological protection but cannot obtain corresponding economic compensation from downstream beneficiaries. More notably, the continuous dominance of economic indicators in local government evaluations further incentivizes jurisdictions to prioritize local interests over regional ecological integrity in trans-boundary contexts, thereby resulting in the phenomenon of ‘patchy’ restoration. Effective ecological governance requires a holistic approach that coordinates entire watersheds and mountains to enhance the connectivity and functional coupling of key ecological nodes. However, these aforementioned institutional barriers restrict the improvement of ecosystem services across broader landscapes.

Reflected in the four challenges discussed above is the essence of ‘growth lessons’ learned during the iterative process of managing large-scale ecological restoration. As the nature-based solutions (NbS) concept matures and cross-regional compensation mechanisms improve, the SSI is poised to become increasingly scientific and sustainable. Drawing from a decade of shared challenges, we propose targeted pathways for future restoration, with the vision of establishing the SSI as a premier model for the UN Decade on Ecosystem Restoration—one that harmonizes economic growth with ecological integrity through NbS.

1. *Operationalizing adaptive management: shifting from static assessment to dynamic evolution.* The core objective is to establish a framework for long-term longitudinal monitoring and a multi-dimensional evaluation indicator system. This will allow for the precise identification of critical thresholds where degraded habitats transition from anthropogenic intervention to autonomous natural succession. To capture the inherent dynamics of ecological recovery, we propose extending SSI evaluation cycles to 15–20 years and integrating ‘space–air–ground’ integrated sensing with long-term ecological research (LTER) networks to track continuous ecological shifts [6]. Furthermore, monitoring indicators must be standardized and categorized at the national level to facilitate consistent, long-term monitoring across all implemented SSI projects. The LTER networks should pivot from a ‘surface-greening bias’ toward longitudinal indicators that reflect true natural succession. Key metrics should include: (i) Soil health: Microbial diversity and soil organic carbon. (ii) Landscape connectivity: Biodiversity corridors and habitat fragmentation indices. (iii) Ecosystem functions: Carbon sequestration, water storage capacity, and nutrient cycling. In tandem with these ecological metrics, socio-economic benefit indicators—such as gross ecosystem product (GEP), GEP-to-gross domestic product (GDP) conversion efficiency, and community livelihood improvements—should be formally integrated into the evaluation system [8]. By synthesizing these biotic, abiotic, and socio-economic indicators, this comprehensive framework provides a rigorous scientific basis for adjusting human interventions in real time, ultimately securing the seamless transition of restored ecosystems toward a self-sustaining state [5].
2. *Refining the NbS concept: toward social–ecological mutualism.* Targeted measures are proposed based on China’s first national guideline for SSI in August 2020, which emphasizes the philosophy of ‘natural restoration first, anthropogenic intervention supplementary measure’ to alleviate some early over-engineering problems. Given funding and resource constraints, we recommend: (i) Adopting ‘trade-off efficiency’ strategies to shift from single-objective to multi-objective optimization, using multicriteria optimization algorithms for optimal resource allocation [5]. (ii) Deepening social–ecological coupling in line with IUCN NbS criteria [9], institutionalizing the free, prior, and informed consent principle, and integrating livelihood security into restoration evaluation systems to consolidate social–ecological resilience.
3. *Advancing bioclimatic-adaptive technological innovation.* Given the complex and diverse geological and climatic environments of China’s ecological protection hotspots, restoration technologies must transition from homogenized engineering to targeted applications. We advocate that, building upon national guidelines, implementers should be encouraged to actively explore locally adapted restoration solutions to achieve synergy between the use of indigenous species for revegetation and micro-topographic modification [10]. Simultaneously, technological applications must be anchored to the core needs of key regions: (i) *Qinghai-Xizang Plateau*: prioritizing enhancing the resilience of alpine meadow ecosystems against permafrost degradation; (ii) *Yellow River Basin*: focusing on regulating the sediment-water nexus and strengthening soil erosion control; (iii) *Yangtze River Economic Belt*: centering on enhancing eco-hydrological connectivity and improving riparian blue carbon sequestration; (iv) *Northern Protective Forest Belt*: focusing on drought-adaptive afforestation to enhance windbreak and sand fixation; and (v) *coastal areas:* considering the protective differences between northern and southern coasts by constructing *mangrove–seagrass–saltmarsh* forest complexes.
4. *Promoting transboundary connectivity and collaborative governance.* We advocate transforming spatial governance to build regional ecological networks across watersheds and mountain ranges [11]. Leveraging habitat corridors to enhance connectivity, we propose adopting the ‘core-corridor-buffer’ matrix—popularized by the Pan-European Ecological Network—to prioritize transboundary ecological corridors within the TZFB framework. Simultaneously, we recommend establishing integrated vertical (central-to-local) and horizontal (inter-jurisdictional) collaborative governance systems. Supported by payment for ecosystem services (PES) based on GEP accounting, this framework balances the interests of upstream conservation zones and downstream beneficiaries by quantifying and monetizing ecological product value (EPV). Encouragingly, national EPV accounting and PES guidelines released between 2021 and 2025 have catalyzed horizontal compensation pilots in key watersheds. This transboundary ecological compensation model offers a scalable reference for global initiatives, including the *Great Green Wall for Restoration and Peace*, the *Trinational Atlantic Forest Pact*, and *Acción Andina*.

The SSI serves as a continental-scale laboratory for pioneering large-scale social–ecological restoration (LSER). By synthesizing the traditional Eastern philosophy of ‘human-nature harmony’ with the adaptivity, reciprocity, and connectivity of global NbS, the SSI has constructed a distinctive NbS-SSI Nexus that localizes and adapts NbS standards to China’s context. Specifically, the SSI offers replicable experience for global LSER in three key aspects: (i) breaking through the limitations of fragmented governance of single elements and establishing a new holistic of a ‘community of life’; (ii) forging a development model that coordinates ecological restoration with livelihood improvement, poverty reduction, and income growth; and (iii) promoting the conceptual shift of restoration assessment from a focus on increasing green coverage to an emphasis on functional enhancement, as well as actively exploring the valorization of ecosystem products through the application of GEP. However, it must be noted that while drawing from these lessons, it is imperative to critically reflect on the portability of the SSI governance model—carefully balancing its universal potential with context-specific limitations. Ultimately, the SSI represents not only a blueprint for the Beautiful China initiative but also strives to become an exemplar for enhancing global biospheric integrity to combat the triple planetary crisis.
